# Industrial Image Anomaly Detection via Synthetic-Anomaly Contrastive Distillation

**DOI:** 10.3390/s25123721

**Published:** 2025-06-13

**Authors:** Junxian Li, Mingxing Li, Shucheng Huang, Gang Wang, Xinjing Zhao

**Affiliations:** 1 School of Information Engineering, Yangzhou Polytechnic College, Yangzhou 225009, China; junxianli@yzpc.edu.cn; 2 School of Electrical and Information Engineering, Jiangsu University JingJiang College, Zhenjiang 212013, China; 3 School of Computer, Jiangsu University of Science and Technology, Zhenjiang 212003, China; schuang@just.edu.cn; 4 School of Computing and Artificial Intelligence, Southwest Jiaotong University, Chengdu 611756, China; eachgood@my.swjtu.edu.cn; 5 Suzhou Zhanchi Tongyang Talent Technology Company, Suzhou 215558, China

**Keywords:** anomaly detection, anomaly localization, knowledge distillation, feature refinement, abnormal synthesis

## Abstract

Industrial image anomaly detection plays a critical role in intelligent manufacturing by automatically identifying defective products through visual inspection. While unsupervised approaches eliminate dependency on annotated anomaly samples, current teacher–student framework-based methods still face two fundamental limitations: insufficient discriminative capability for structural anomalies and suboptimal anomaly feature decoupling efficiency. To address these challenges, we propose a Synthetic-Anomaly Contrastive Distillation (*SACD*) framework for industrial anomaly detection. *SACD* comprises two pivotal components: (1) a reverse distillation (RD) paradigm whereby a pre-trained teacher network extracts hierarchically structured representations, subsequently guiding the student network with inverse architectural configuration to establish hierarchical feature alignment; (2) a group of feature calibration (*FeaCali*) modules designed to refine the student’s outputs by eliminating anomalous feature responses. During training, *SACD* adopts a dual-branch strategy, where one branch encodes multi-scale features from defect-free images, while a Siamese anomaly branch processes synthetically corrupted counterparts. *FeaCali* modules are trained to strip out a student’s anomalous patterns in anomaly branches, enhancing the student network’s exclusive modeling of normal patterns. We construct a dual-objective optimization integrating cross-model distillation loss and intra-model contrastive loss to train *SACD* for feature alignment and discrepancy amplification. At the inference stage, pixel-wise anomaly scores are computed through multi-layer feature discrepancies between the teacher’s representations and the student’s refined outputs. Comprehensive evaluations on the MVTec AD and BTAD benchmark demonstrate that our method is effective and superior to current knowledge distillation-based approaches.

## 1. Introduction

In contemporary industrial production, product quality assurance serves dual imperatives: maintaining a competitive advantage and ensuring consumer trust. The integration of automation and intelligent systems has elevated industrial visual anomaly detection to a pivotal role in optimizing manufacturing precision and operational throughput [1,2,3]. Nevertheless, conventional supervised learning paradigms encounter practical constraints in this domain, particularly stemming from the labor-intensive process of collecting and labeling defective visual data, compounded by data privacy regulations that further restrict anomaly sample availability. These operational challenges necessitate alternative methodologies for precise defect identification. Unsupervised learning has consequently emerged as a promising solution, demonstrating notable efficacy in scenarios with limited annotated data [2].

Within industrial visual inspection frameworks, unsupervised techniques enable anomaly detection and localization model development through the exclusive utilization of defect-free training samples. This paradigm shift addresses both the scarcity of anomalous exemplars and the prohibitive costs associated with manual annotation. Standard implementations employ pristine image datasets for model training, while evaluation protocols incorporate mixed sets containing both normal and anomalous instances. Current methodological approaches predominantly frame this challenge as an out-of-distribution detection problem. Recent advancements in unsupervised anomaly detection have yielded several principal technical trajectories, including feature embedding-based methods [4,5,6,7], reconstruction-based methods [8,9,10].

Reconstruction-based approaches identify anomalies through differential error analysis between original and reconstructed images [10,11]. The underlying hypothesis posits that autoencoders or generative adversarial networks achieve superior reconstruction fidelity on nominal patterns compared to anomalous regions. However, practical implementations reveal limitations in discriminative capacity as reconstruction artifacts may obscure differences between normal and defective regions [7,12]. This inherent ambiguity frequently manifests as performance instability across diverse industrial use cases.

Recent advancements in teacher–student frameworks have demonstrated notable efficacy in visual anomaly detection through hierarchical feature reconstruction. Bergmann et al. [4] pioneered this paradigm with their Uninformed Students, establishing foundational work in unsupervised anomaly recognition via teacher–student discrepancy analysis. Subsequent innovations [5,13] improve the model through systematic multi-layer feature alignment, establishing standardized protocols for multi-resolution anomaly mapping via cross-layer matching errors between architecturally symmetric teacher–student pairs.

While achieving benchmark performance, contemporary teacher–student-based anomaly detection approaches reveal several critical limitations. First, the absence of regularization mechanisms during knowledge transfer makes student model overfitting risks. Empirical evidence suggests that even when trained exclusively on anomaly-free samples, student networks may inadvertently encode anomalous patterns through latent feature sharing with teacher representations, thereby compromising detection specificity. Second, existing implementations [14,15,16] frequently employ computationally intensive architectures to enhance accuracy, exemplified by AEKD’s dual-student configuration [14], which escalates hardware requirements and complicates industrial deployment.

To overcome these constraints, we propose a Synthetic-Anomaly Contrastive Distillation (*SACD*) framework. The *SACD* framework comprises several crucial components: (1) a reverse distillation mechanism where a frozen teacher model utilizing ImageNet-pretrained weights extracts multi-scale representations to guide a structurally inverted student network through layer-wise feature alignment, and (2) a set of feature calibration (*FeaCali*) modules that eliminate noise interference while retaining critical anomaly indicators in student-decoded features. This configuration preserves normal pattern learning while proactively exposing the student model to synthetic anomalies, thereby enhancing discriminative capacity against potential defects. During training, *SACD* achieves dual objectives. On the one hand, it can reinforce inter-model consistency in normal feature representation through contrastive distillation, and on the other hand, we intend to amplify feature discrepancies in abnormal pattern decoding via synthetic anomaly confrontation. Moreover, *FeaCali* modules are designed to ensure parameter-efficient alignment between student and teacher embeddings while mitigating overfitting risks.

The proposed framework employs a dual-objective loss function that orchestrates the training process through two aspects: cross-model feature alignment between the student–teacher pair and intra-model component coordination within the Siamese students. This composite loss simultaneously enforces representation consistency across network hierarchies while maintaining functional complementarity among different streams, thereby ensuring concurrent optimization of normal pattern reconstruction fidelity and abnormal feature discrimination capability. During inference, pixel-wise anomaly scores are computed via multi-scale feature deviation metrics between the teacher’s preserved knowledge and the student’s calibrated outputs. Extensive benchmark evaluations on the MVTec AD dataset [17] demonstrate the effectiveness of our proposed *SACD* framework. Qualitative and quantitative results show that *SACD* achieves superior performance in anomaly detection and localization tasks, surpassing current baseline methods in terms of detection accuracy, while achieving optimizations in model size and computational complexity.

In summary, the main contributions of this paper are as follows:

- We propose a novel Synthetic-Anomaly Contrastive Distillation framework for industrial image anomaly detection, which enhances anomalous feature decoupling while preserving normal pattern reconstruction capabilities of the student model.
- We construct a dual-objective loss function encompassing both cross-model feature alignment and intra-model component coordination, enabling hierarchical representation consistency and discrepancy amplification between the teacher–student model.
- Extensive experiments conducted on the MVTec AD and BTAD dataset demonstrated our proposed method is effective and achieves superior anomaly detection performance with optimized model size and computational efficiency compared with the current KD-based approaches.

## 2. Related Works

Prior to extensive adoption of deep learning methodologies, industrial anomaly detection techniques progressively advanced into two dominant paradigms. One approach emphasizes the identification of discrepancies by means of feature embedding, whereas the other employs reconstruction-based mechanisms to detect anomalies.

### 2.1. Feature Embedding-Based Methods

Methods based on feature embedding commonly employ architectures such as VGG [13], ResNet [7], or Vision Transformer [18], utilizing intermediate or final feature representations for anomaly detection and localization. In the absence of anomalous samples during training, some approaches generate synthetic anomalies from normal images to train detection models. For instance, PatchSVDD [19] segments images into uniform patches for model training, improving the detection of localized anomalies. MOCCA [20] employs autoencoders to extract multi-scale features and delineate normal feature boundaries at each layer. Similarly, CutPaste [21], a notable data augmentation method, synthesizes anomalies by cutting and pasting regions of normal images, enabling the network to differentiate between normal and anomalous patterns. Some methods have mapped feature embeddings into distribution maps to appropriate distributions for anomaly identification [22,23,24,25]. For example, CS-Flow [23] enhanced performance by incorporating cross-convolution blocks into normal flow, leveraging contextual information across scales. Similarly, CFlow-AD [24] introduced positional encoding into conditional normal flow, analyzing the rationale behind the multivariate Gaussian assumption and normal flow’s efficiency.

To improve detection, some methods store normal image embeddings in a memory bank for inference comparison. K-Nearest Neighbors (KNNs), widely used in unsupervised anomaly detection, operates at the sample level. Inspired by KNN, SPADE [26] employs multi-resolution feature pyramids for pixel-level anomaly segmentation. PaDim [27] models the normal class probabilistically using multivariate Gaussians, with memory bank size dependent on image resolution rather than dataset size. However, its scalability is limited by computational demands for large CNNs. PatchCore [28] significantly advanced industrial AD, excelling on the MVTec AD dataset. It uses secondary sampling of a core set to optimize memory bank usage, reducing inference costs while maintaining high performance. Anomalies are detected by measuring distances between test samples and memory bank features. Lee et al. [29] proposed CFA, adapting features via coupled hyperspheres. Using transfer learning, it determines anomalies based on the positional relationship between test features and hypersphere surfaces in the memory bank.

Knowledge distillation methods leverage representation discrepancies between the teacher and student model for anomaly detection, demonstrating strong effectiveness. Uninformed Students [4] pioneered its application in industrial image anomaly detection, achieving notable results. Subsequent works, such as STPM [5] and MKD [13], advanced multi-scale feature transfer across model layers, albeit using different pre-trained architectures. MKD further revealed that a lighter student network outperforms one mimicking the teacher’s structure. RD4AD [12] introduced reverse distillation into the teacher–student pair with multi-scale feature fusion and bottleneck embeddings to reduce redundancy, enabling efficient feature reconstruction. AST [30] addressed the issue of similar feature extraction for anomalies by proposing an asymmetric architecture, complemented by normalization flows to mitigate structural inconsistencies and estimation bias. Overfitting due to capacity mismatches and knowledge transfer limitations has been a challenge in prior methods. To mitigate this, some methods proposed to incorporate extra auxiliary measures, such as memory bank [31,32], feature consistency [33,34,35,36,37], segmentation task [38,39], etc.

### 2.2. Reconstruction Based Methods

Reconstruction-based methods train encoders and decoders to reconstruct images for anomaly detection, reducing reliance on pre-trained models while enhancing anomaly detection capabilities. However, their limited ability to extract high-level semantic features often results in suboptimal image classification performance. Autoencoders [8], generative adversarial networks [40], and difussion models [41] are widely used for this purpose, with most reconstruction networks comprising encoder–decoder architectures [8,9,42]. For example, DRAEM [8] exemplifies reconstruction-based techniques by synthesizing anomalous images using external datasets and reconstructing them into normal images, improving generalization. It further integrates original and reconstructed images into a segmentation network to predict anomalous regions, boosting anomaly segmentation accuracy. Nevertheless, DRAEM faces challenges with near-distribution anomalies. Yan et al. [9] introduced MLIR, a multi-level image reconstruction framework that treats reconstruction as a denoising task across resolutions, enabling the detection of both global structural and fine-grained anomalies. DSR [11] employs quantized feature space representation and dual decoders, avoiding image-level anomaly synthesis. By sampling the quantized feature space, DSR generates near-distribution anomalies in a controlled manner. NSA [10] achieves superior performance without external data by leveraging diverse augmentation techniques, outperforming prior methods reliant on additional datasets.

## 3. Method

**Figure 1:**
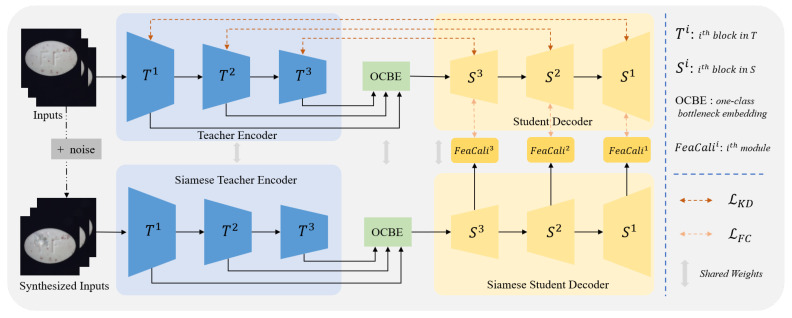
Overview of our proposed *SACD* framework.

In this section, we elaborate on the overall framework of the proposed Synthetic-Anomaly Contrastive Distillation (*SACD*) framework, as illustrated in Figure 1. *SACD* comprises a Siamese reverse distillation flow and a group of feature calibration modules. During training, the teacher encoder is frozen; the weights of the one-class bottleneck embedding (*OCBE*) module, student decoder, and *FeaCali* modules are optimized via a dual-objective loss function.

### 3.1. Reverse Distillation

Reverse distillation comprises three main components: the teacher encoder, the *OCBE* module, and the student decoder.

#### 3.1.1. Teacher Encoder

In unsupervised anomaly detection using knowledge distillation, constructing a robust teacher model is essential. Following Wang et al. [33], we employ a WideResNet-50 model pre-trained on ImageNet as the teacher encoder. The ResNet-like architecture, with its multiple residual blocks, enables the network to capture complex feature representations. Intermediate outputs from these blocks are treated as multi-scale feature representations of input images [5,14,33,43,44]. Based on this idea, and in line with previous work, by default, feature maps from the 1st, 2nd, and 3rd layers of WideResNet-50 are selected as learning targets for the student model. This allows the student decoder to inherit rich multi-level features, enhancing multi-scale feature reconstruction and anomaly perception.

Let *T* denote the teacher model. For an input *I*, the multi-scale features are obtained as

(1) $$f_{1}^{t},f_{2}^{t},f_{3}^{t}=T\left(I\right),$$

where $f_{1}^{t}$, $f_{2}^{t}$, and $f_{3}^{t}$ correspond to the outputs of the 1st, 2nd, and 3rd layers of the teacher encoder *T*, respectively.

#### 3.1.2. One-Class Bottleneck Embedding

**Figure 2:**
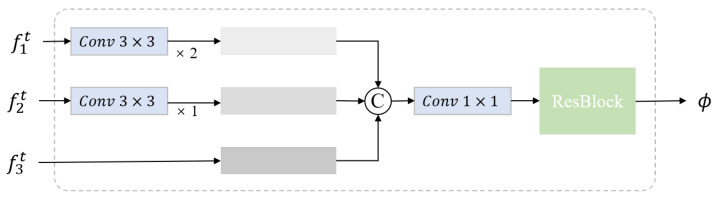
Architecture of the OCBE module.

In conventional encoder–decoder frameworks, the decoder typically relies on the final-layer output of the encoder. However, reverse distillation poses challenges when transferring high-level embeddings from the teacher encoder directly to the student decoder, as it hinders the reconstruction of fine-grained features. To mitigate this, as is presented in Figure 2, OCBE is designed to facilitate multi-scale feature fusion and dimensionality reduction through feature compaction, preserving essential information while mapping high-dimensional inputs to a lower-dimensional space. This design enhances the student decoder’s ability to interpret hierarchical features from the teacher, leading to more expressive and efficient reconstructions.

Let Φ denote the fusion and compression function of the OCBE module. By integrating the three feature embeddings $f_{1}^{t}$, $f_{2}^{t}$, and $f_{3}^{t}$, the resultant representation can be expressed as ϕ:

(2) $$\phi =\Phi (f_{1}^{t},f_{2}^{t},f_{3}^{t}).$$

#### 3.1.3. Student Decoder

Upon receiving the compressed multi-scale teacher features ϕ, the student decoder is required to reconstruct feature embeddings of identical dimensions across different scales based on these features. Consequently, in the reverse distillation process, the student decoder is designed with an inverse architecture, while ensuring that the size of its output tensors remains consistent with the corresponding teacher embeddings. Specifically, the number of forward residual blocks in the student decoder is aligned with that of the teacher encoder, while the channel dimensions are adjusted via upsampling to match the embedding dimensions of the teacher model at the corresponding scales. It is worth noting that, in contrast to conventional convolutional pooling operations, the reverse process employs deconvolution for upsampling. The entire procedure can be formalized as follows:

(3) $$f_{3}^{s},f_{2}^{s},f_{1}^{s}=S\left(\phi \right),$$

where $f_{3}^{s}$, $f_{2}^{s}$, and $f_{1}^{s}$ correspond to the outputs of the 1st, 2nd, and 3rd layers of the student decoder *S*, respectively.

### 3.2. Siamese Reverse Distillation Flow

In traditional multi-scale feature knowledge distillation frameworks, teacher–student models typically follow a one-to-one configuration, where the teacher model transfers multi-layer embedding knowledge of normal images to the student model. The student then identifies anomalous regions based on the representation differences between the teacher and student model. However, during training, the student model may overfit or develop overly strong encoding capabilities, causing it to produce representations for anomalous regions that closely resemble those of the teacher, thereby degrading anomaly detection performance. In view of this, we extend the original anomaly-free input and synthesize abnormal substitutes of normal samples using image-level anomaly synthesis. Additionally, we introduce a Siamese reverse distillation flow to encode and decode the features of both normal and synthesized abnormal inputs.

#### 3.2.1. Normal RD Branch

Assuming the input anomaly-free sample is denoted as $I_{n}$, the processing through the normal branch yields two sets of features:

(4) $$f_{1}^{t_{n}},f_{2}^{t_{n}},f_{3}^{t_{n}}=T\left(I_{n}\right),$$

(5) $$f_{3}^{s_{n}},f_{2}^{s_{n}},f_{1}^{s_{n}}=S\left(\Phi \left(f_{1}^{t_{n}},f_{2}^{t_{n}},f_{3}^{t_{n}}\right)\right),$$

where $f_{1}^{t_{n}}$, $f_{2}^{t_{n}}$, and $f_{3}^{t_{n}}$ denote the anomaly-free features encoded by the teacher model, while $f_{3}^{s_{n}}$, $f_{2}^{s_{n}}$, and $f_{1}^{s_{n}}$ represent the feature embeddings reconstructed by the student decoder.

#### 3.2.2. Abnormal RD Branch

As described above, normal branch is responsible for receiving and encoding multi-scale features of normal images. In this way, abnormal branch receives and encodes the anomalous version (synthesized) of the corresponding normal image. Importantly, our goal is for the student network to exclusively encode features from the normal regions, while ensuring a significant difference in the representation of anomalous regions between the teacher and student models. In addition to the basic RD model, the anomaly branch also incorporates an anomaly synthesis module and a feature refinement module. The details of these components are described in the following subsections.

(1) Anomaly Synthesis

**Figure 3:**
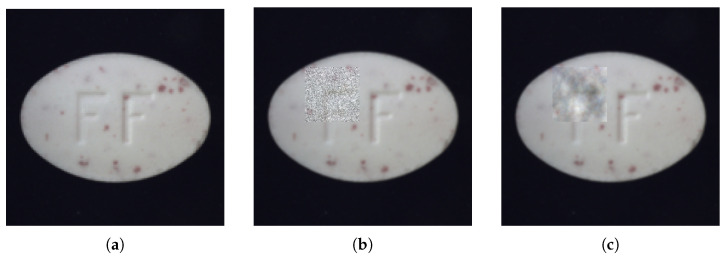
Visualization of original image and various pseudo-anomaly injections. (**a**) Original anomaly-free image; (**b**) pseudo-anomaly image with Gaussian noise; (**c**) pseudo-anomaly image with Simplex noise.

This paper assumes that normal and anomalous patterns might share some basic features, which allows the student model to reconstruct anomalies effectively. To handle this, we create anomalous versions of normal images during training. This helps the student model learn about anomalies beforehand, preparing it for better detection later. Recent studies have suggested methods like Gaussian noise [33], masking [45], and CutPaste [21] for creating anomalies. Given its effectiveness and simplicity, we use the simplex method as our default approach. Simplex noise performs better than Gaussian noise when simulating anomalies using a power-law distribution. As shown in Figure 3, simplex noise creates more natural-looking anomalies compared to Gaussian noise. Let $I_{a}$ represent the synthesized anomalous image, and the full process is described in Algorithm 1.

Consistent with the normal branch, the RD model encodes the synthesized anomaly samples into two sets of features:

(6) $$f_{1}^{t_{a}},f_{2}^{t_{a}},f_{3}^{t_{a}}=T\left(I_{a}\right),$$

(7) $$f_{3}^{s_{a}},f_{2}^{s_{a}},f_{1}^{s_{a}}=S\left(\Phi \left(f_{1}^{t_{a}},f_{2}^{t_{a}},f_{3}^{t_{a}}\right)\right),$$

where $f_{1}^{t_{a}}$, $f_{2}^{t_{a}}$, and $f_{3}^{t_{a}}$ denote the anomaly-free features encoded by the teacher model, while $f_{3}^{s_{a}}$, $f_{2}^{s_{a}}$, and $f_{1}^{s_{a}}$ represent the feature embeddings reconstructed by the student decoder.

Since the input consists of anomaly samples, the features encoded by the teacher model will contain anomalous patterns. After undergoing fusion and compression operations in the OCBE module, these features may still retain latent anomalous patterns due to the absence of an explicit mechanism to eliminate such anomalies. Consequently, the multi-scale feature embeddings reconstructed by the student model will also include latent anomaly information, which can undermine the feature matching process between the teacher and student models, thereby degrading the performance of anomaly detection.
**Algorithm 1:** Pseudo-code of the process of anomaly synthesis **Input**: Normal training set χ, discrete range U[a,b], noise parameter λ **Output**: Modified training set with Simplex noise  

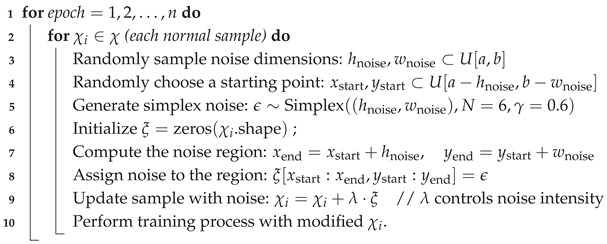

(2) Feature Calibration Module

**Figure 4:**
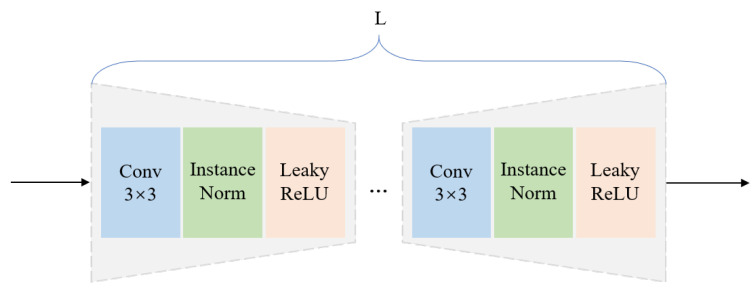
Structure of the *FeaCali* module.

As stated in the assumption, when the input image contains anomalies, the feature calibration (*FeaCali*) module is designed to filter out potential anomalous patterns from the student decoder’s outputs. This prevents performance degradation caused by anomaly leakage. To keep the module lightweight, *FeaCali* uses stacked convolutional blocks in a bottleneck style, including convolution, InstanceNorm, and LeakyReLU layers, where the channel dimensionality is halved progressively at each layer. After obtaining restored feature embeddings $f_{1}^{t_{a}}$, $f_{2}^{t_{a}}$, and $f_{3}^{t_{a}}$, the *FeaCali* module operates in multi-scale modes, denoted as $FeaCali^{1}$, $FeaCali^{2}$, and $FeaCali^{3}$, individually. Its main task is to take intermediate layer outputs as input and refine them by augmenting normal features. Through this process, $f_{1}^{t_{a}}$, $f_{2}^{t_{a}}$, and $f_{3}^{t_{a}}$ are refined into $f_{1}^{t_{n}^{\prime }}$, $f_{2}^{t_{n}^{\prime }}$, and $f_{3}^{t_{n}^{\prime }}$. Figure 4 shows the structure of the MFR module. In the experiments, *L* is set to 2.

### 3.3. Training Objective

We design a dual-objective loss function to train the OCBE module, student decoder, and *FeaCali* module. This function ensures consistent hierarchical representations between the teacher and student models while amplifying discrepancies for anomaly detection. The total loss consists of two parts: cross-model feature alignment loss, denoted as $L_{1}$, and intra-model component coordination loss, denoted as $L_{2}$. $L_{1}$ transfers knowledge from the teacher model to the student model, enabling the student to replicate the teacher’s understanding of normal image features. $L_{2}$ optimizes the *FeaCali* module, helping it filter out potential anomalies and reconstruct high-quality normal features. The overall loss is expressed as

(8) $$L_{total}=L_{1}+\alpha L_{2},$$

where α is a positive regularization parameter for adjusting the optimization weights of the *FeaCali* module.

#### 3.3.1. Intra-Model Component Coordination Loss

After building the *FeaCali* module, we aim for it to enhance normal features and filter out anomalous ones. A simple approach is to use the multi-scale restored feature embeddings from the student decoder’s normal branch as ground truth to guide feature reconstruction. The loss $L_{2}$ is computed using cosine similarity to optimize the *FeaCali* module.

First, the features $f_{1}^{s_{n}^{\prime }}$, $f_{2}^{s_{n}^{\prime }}$, and $f_{3}^{s_{n}^{\prime }}$ are flattened from shape (B, C, H, W) to (B, C×H×W) to calculate the similarity between teacher and student features at each layer. The total loss is the sum of these layer-wise losses:

(9) $$L_{2}=1-\sum_{k=1}^{K}\frac{\psi {\left(f_{k}^{s_{n}}\left(h,w\right)\right)}^{⊤}\cdot \psi \left(f_{k}^{s_{n}^{\prime }}\left(h,w\right)\right)}{∥\psi \left(f_{k}^{s_{n}}\left(h,w\right)\right)∥∥\psi \left(f_{k}^{s_{n}^{\prime }}\left(h,w\right)\right)∥},$$

where ψ(·) represents the flattening operation, K∈{1,2,3} is the number of feature layers used in training, and h,w denote the height and width of the k-th feature map. Cosine similarity measures feature similarity, and minimizing $L_{2}$ optimizes the *FeaCali* module.

#### 3.3.2. Cross-Model Feature Alignment Loss

In order to effectively transfer the teacher model’s multi-scale knowledge to the student model, the teacher–student models employ cosine similarity as the knowledge distillation loss $L_{1}$ for knowledge transfer and feature alignment. The loss function for optimizing the *OCBE* module and student decoder is derived from the following equation:

(10) $$L_{1}=1-\sum_{k=1}^{K}\left\{\frac{{\left(f_{k}^{t_{n}}\left(h,w\right)\right)}^{⊤}\cdot \left(f_{k}^{s_{n}}\left(h,w\right)\right)}{\|f_{k}^{t_{n}}\left(h,w\right)\|\|f_{k}^{s_{n}}\left(h,w\right)\|}\right\},$$

where, K∈{1,2,3} denotes the number of feature layers used in training, and *h* and *w* represent the height and width of the k-th feature map.

### 3.4. Inference and Anomaly Scoring

During the inference stage, the entire reverse distillation process is fully retained, and the output of the student decoder is enhanced by the *FeaCali* module for normal feature refinement. Given a query image $I_{\mathrm{query}}$, we obtain a pair of multi-level feature embeddings from the teacher and student models, denoted as $≺f_{1}^{t_{q}}$, $f_{2}^{t_{q}}$, $f_{3}^{t_{q}}≻$ and $≺f_{1}^{s_{q}^{\prime }}$, $f_{2}^{s_{q}^{\prime }}$, $f_{3}^{s_{q}^{\prime }}≻$, respectively. Then we calculate the anomaly score based on the difference between the outputs of the intermediate layers at corresponding positions of the teacher and student models:

(11) $$M_{k}^{h,w}=1-\frac{{\left(f_{k}^{t_{q}}\left(h,w\right)\right)}^{⊤}\cdot \left(f_{k}^{s_{q}^{\prime }}\left(h,w\right)\right)}{\|f_{k}^{t_{q}}\left(h,w\right)\|\|f_{k}^{s_{q}^{\prime }}\left(h,w\right)\|},$$

where $M_{k}^{h,w}$ refers to the anomaly score in (*h*, *w*) of the k-th feature map, *h* and *w* are the corresponding location in the query image.

**Figure 5:**
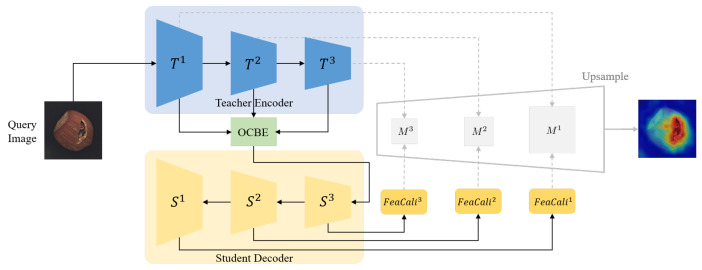
Inference pipeline of our proposed method.

To locate anomalies in the query image, we combine anomaly prediction score maps from different scales. Intermediate layer features are upsampled to match the query image’s resolution using bilinear interpolation. The final anomaly map is computed as

(12) $$M=\sigma \left(\sum_{i=1}^{K}\mathrm{up}\_\mathrm{sample}\left(M_{k}\right)\right),$$

where M represents the final anomaly map, and σ(·) is a Gaussian filter with σ=4 to smooth noise in the map. The inference process is illustrated in Figure 5.

## 4. Experiment Setting and Results

### 4.1. Dataset

To evaluate the performance of the proposed method for industrial anomaly detection, experiments were conducted on the widely-used MVTec AD benchmark [17] and BTAD dataset [46]. The MVTec AD dataset includes 15 sub-datasets with a total of 5354 images, of which 1725 are in the test set. Each sub-dataset consists of training data containing only normal samples and test sets that include both normal and anomalous samples. The test sets also provide various defect types along with corresponding ground truth masks for anomalies. The BTAD dataset is a practical benchmark for industrial anomaly detection, comprising 2830 images collected from real-world scenarios involving three types of industrial products. These samples exhibit various structural and surface-level defects.

### 4.2. Evaluation Metrics

The performance of image-level anomaly detection is evaluated using the area under the receiver operating characteristic curve (AUROC) based on generated anomaly scores. Following prior work, we calculate the class-average AUROC on the MVTec dataset. For segmentation performance, we use pixel-wise AUROC and the PRO metric. The PRO score measures the overlap and recovery of connected anomaly components, providing a more accurate evaluation for anomalies of varying sizes.

### 4.3. Implementation Details

Following prior works, anomaly detection and localization are performed on one class at a time. Consistent with previous studies [12,14], we use a WideResNet50 pre-trained on ImageNet as the teacher model, while the student model and MRF module parameters are randomly initialized. The outputs of the 1st, 2nd, and 3rd ResBlocks of WideResNet50 are selected as multi-scale anomaly representations. As in [7,12,14], images are resized to 256 × 256. No data augmentation is applied during experiments. For anomaly synthesis, the parameter λ is set to 0.4 by default, and only half of each batch is used for pseudo-anomaly synthesis. And the parameters for Simplex noise generation are set by default as follows: octaves = 3 to introduce multi-scale texture variation, persistence = 0.5 to balance detail retention and smoothness, and frequency = 32.0 to control the granularity of the generated anomalies. In addition, the width and height of each noise region are randomly sampled within the range of (0, 0.5) of the corresponding image’s dimensions to simulate variable anomaly shapes. Equilibrium coefficient α in loss function is set to 0.2. The model is trained using the Adam optimizer with β=(0.5,0.999), a learning rate of 0.001, and a batch size of 16 for 200 epochs. A Gaussian filter with σ=4 is applied to smooth the anomaly score map.

### 4.4. Experimental Results

#### 4.4.1. Results on MVTec AD

We compare our method with representative state-of-the-art algorithms, including Uninformed Students [4] (US), MKD [13], Patch-SVDD [19], SPADE [26], PaDiM [27], CutPaste [21], RIAD [47], RD4AD [12], MaMiNet [48], MMR [49], AEKD [14], and MSFR [16]. The results for industrial image anomaly detection and localization are presented in Table 1 and Table 2, respectively.

Table 1 compares our method with recent approaches for anomaly detection. Clearly, our method achieves the best average performance in detecting texture and object anomalies, reaching 99.9% and 99.1%, respectively, surpassing the sub-optimal method AEKD by 0.2% and 0.6%. Overall, our method achieves the highest anomaly detection AUROC of 99.3% across all instances. Additionally, it attains 100% detection rates for categories such as *carpet*, *grid*, *leather*, *bottle*, and *toothbrush*. These results highlight the effectiveness and superiority of our approach.

Compared to the native RD4AD method, *SACD* improves normal feature reconstruction by post-processing the student decoder’s output, reducing potential abnormal expressions. This enhances the consistency of normal feature representations and the distinction of abnormal regions between the teacher and student models. By incorporating prior anomaly pattern learning during training, the student network is better equipped to augment itself, strengthening the alignment and differentiation between the teacher and student models.

Table 2 compares the proposed method with others for anomaly localization, with each entry showing pixel-level AUROC and PRO (pixel-level AUROC/PRO). Overall, our method achieves the best results, with an average AUROC of 98.3% and PRO of 94.8% across all instances. Specifically, for pixel-level AUROC, SPADE, PaDiM, RIAD, CutPaste, RD4AD, MMR, MSFR, and AEKD are 1.8%, 0.8%, 4.1%, 0.5%, 0.2%, 1.1%, 1.0%, and 0.2% lower than ours, respectively. For PRO, SPADE, PaDiM, RD4AD, MMR, MSFR, and AEKD are 3.1%, 2.7%, 0.8%, 2.2%, 1.7%, and 0.8% lower, respectively.

**Figure 6:**
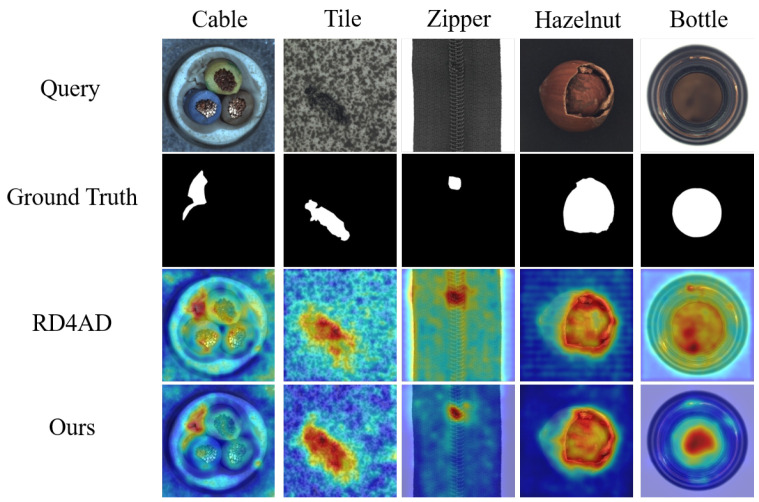
Comparative visualization of anomaly detection methods on representative cases.

For texture anomaly detection, our method achieves 98.2% AUROC and 95.7% PRO on average. For object categories, it achieves 98.4% AUROC and 94.4% PRO. Notably, our method outperforms others in anomaly localization across nearly all categories. Compared to RD4AD, *SACD* shows improved localization performance. Figure 6 provides visual comparisons for representative textures and objects. Both qualitative and quantitative analyses confirm the effectiveness and advancement of our method. For pixel-level anomaly detection, enhancing representation consistency and differences between teacher and student models in the knowledge distillation framework is crucial. *SACD* improves normality in student outputs by introducing a forged anomaly prior and a group of lightweight feature calibration *FeaCali* module during training, boosting the accuracy of anomaly localization in the reverse distillation model.

#### 4.4.2. Results on BTAD

Table 3 compares the pixel-level anomaly localization performance of our proposed method with several representative approaches, including VT-ADL, FastFlow, Patch-SVDD, and RD4AD, on the BTAD dataset. As shown by the experimental results, our method achieves the best average performance across all three product classes, with a pixel-level AUROC of 97.9%, and ranks first on Class02 and Class03, reaching 97.4% and 99.8%, respectively.

Compared to the baseline reverse distillation method RD4AD, our proposed *SACD* framework enhances the student decoder with a multi-scale feature calibration module, and incorporates an abnormality synthesis strategy alongside an inter-model component consistency loss during training. These enhancements enable the model to deliver more accurate anomaly localization during inference, especially when processing defective samples. These results demonstrate the effectiveness and superiority of the proposed method.

### 4.5. Structural and Parametric Analysis

This section primarily investigates the impact of anomaly synthesis intensity, different anomaly synthesis modes, the equilibrium coefficient α in the loss function, the structure of the *FeaCali* module, and the dependency on the pre-trained model on the proposed method’s performance. Throughout the experiments, other hyperparameters were kept at their default settings and remained fixed.

#### 4.5.1. Complexity Analysis

To provide concrete evidence of *SACD*’s computational efficiency, especially in comparison with the dual-student AEKD baseline, we report the model complexity and runtime performance under various *FeaCali* depths *L*. As shown in Table 4, *SACD* achieves consistently strong performance across different settings, with all configurations maintaining inference latency under 4 ms and parameter counts below 166 M.

Interestingly, increasing the *FeaCali* depth from L=1 to L=2 results in a slight reduction in parameters (from 165.08 M to 158.9 M) and FLOPs (from 43.3 G to 39.67 G), while the AUROC improves from 99.2% to 99.3%. Further increasing *L* to 3 or 4 does not yield additional accuracy gains and may even lead to a slight degradation (e.g., 99.0% at L=4), while keeping complexity similar. These observations suggest that L=2 represents the optimal trade-off point, i.e., the accuracy–efficiency knee, where the model achieves peak performance with minimal computational cost.

In contrast, the AEKD baseline, despite having a lower parameter count (132.92 M), shows higher inference latency (4.64 ms) and lower accuracy (98.9%), likely due to the overhead from its dual-student architecture. This confirms that *SACD* not only offers superior anomaly detection performance but also exhibits better computational efficiency, particularly at L=2, validating the design of the *FeaCali* module.

#### 4.5.2. Effect of Pseudo-Anomaly’s Intensity

**Figure 7:**
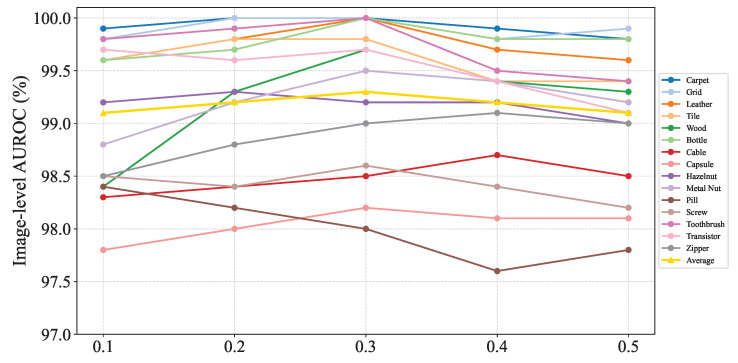
Influence of various pseudo-anomaly’s intensities.

In real industrial scenarios, product defects vary in severity, but all defective products are classified as faulty. To simulate this, we adjusted the anomaly intensity in the synthesis method to evaluate the model’s performance in anomaly detection and localization under varying intensities. Figure 7 compares the model’s performance across different anomaly intensities. The results indicate that when the synthetic anomaly intensity λ is set to 0.3, the model demonstrates superior generalization for both anomaly detection and localization. However, for specific products like *zipper*, the model performs better with an anomaly intensity of 0.4. The optimal intensity can be selected based on task-specific requirements.

#### 4.5.3. Comparative Study of Anomaly Synthesis Methods

There are various approaches to anomaly synthesis, and as discussed in Section 3.2.2, we focus on concise image-level anomaly synthesis strategies, including CutPaste, Gaussian noise, and Simplex noise. Table 5 presents the comparative results of these methods. It is evident from the results that Simplex noise achieves the best performance among the three methods, owing to its ability to generate more natural-looking synthetic anomalies. In contrast, CutPaste and Gaussian noise demonstrate comparable performance, with no significant distinction between them.

#### 4.5.4. Influence of the Equilibrium Coefficient on Loss Function Optimization

As outlined in Section 3.3, α is a hyperparameter that controls the weight of the intra-model component coordination loss ($L_{2}$) in the total loss function. A higher α value increases the influence of $L_{2}$ on the model optimization. To determine the optimal balance, we conducted several ablation experiments on α to identify the point where the model achieves the best performance trade-off. Table 6 presents the anomaly detection and localization results for different α values.

The results show that, as α increases, the model’s performance gradually decreases. This indicates that the cross-model feature alignment loss plays a crucial role in enabling the student network to effectively learn multi-layer image feature embeddings and should dominate the loss function. In contrast, the intra-model component coordination loss is less effective at filtering anomalous information from normal features and enhancing normal feature reconstruction. From Table 6, it is evident that the model performs best when α is set to 0.2, achieving an optimal balance between the two losses during training.

#### 4.5.5. Effect of *FeaCali* Module Configuration

(1) Effect of *FeaCali* Depth

**Figure 8:**
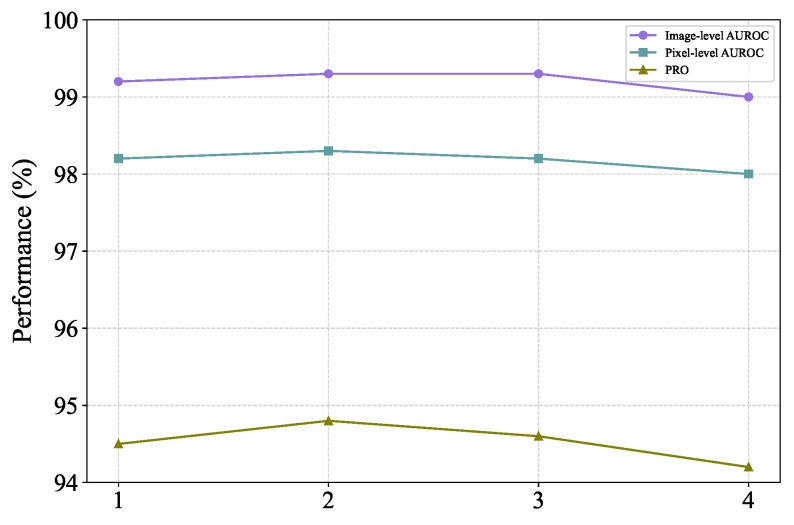
Comparison of model detection performance at different depths of *FeaCali* module.

The *FeaCali* module is composed of stacked bottleneck layers. In this subsection, we investigate the impact of varying the depth of this module on the model’s performance, as illustrated in Figure 8. As the depth of the module increases gradually, the overall anomaly detection performance of the model initially improves, reaches a plateau, and subsequently begins to decline. Based on the experimental results, it can be inferred that setting the depth to 2 strikes a good balance between performance and complexity, making it a suitable choice.

(2) Selection of Normalization/Activation Function

**Figure 9:**
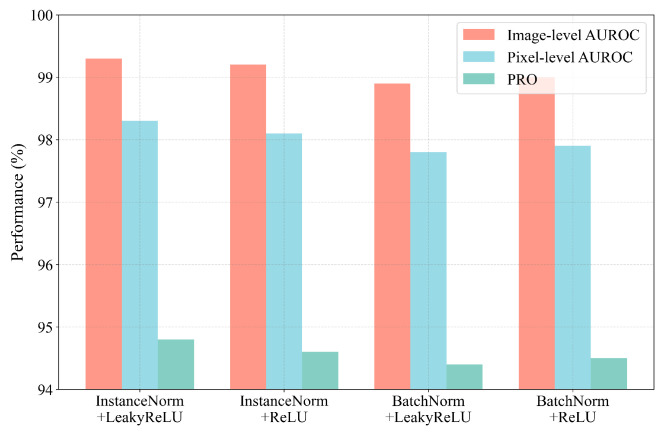
*FeaCali* module with different combinations of normalization/activation functions.

Figure 9 compares the performance of different normalization and activation function combinations in unsupervised anomaly detection tasks. It is observed that the combination of Instance Normalization and LeakyReLU consistently outperforms the others, primarily due to its superior ability to preserve local anomalous features and enhance sensitivity to subtle deviations. InstanceNorm normalizes each sample independently, thereby avoiding the inference instability associated with BatchNorm’s reliance on batch-level statistics—an issue especially prominent in small-batch or single-sample inference scenarios. Meanwhile, LeakyReLU alleviates the dead neuron problem inherent in ReLU and retains non-zero gradients in the negative domain, facilitating the modeling of fine-grained anomalies. As a result, this configuration demonstrates greater robustness and improved performance on both image-level and pixel-level evaluation metrics. Accordingly, it is adopted as the default setup for the *FeaCali* module.

#### 4.5.6. Dependency on Pretrained Model

Anomaly detection methods based on the teacher–student framework rely on powerful pre-trained models, where the teacher model’s generalization capability is critical to performance. Table 7 presents the anomaly detection results of our framework using various ResNet architectures as pre-trained models. The results show that WideResNet50, by expanding network width, significantly enhances encoding capacity and adaptability to diverse data distributions compared to traditional ResNet architectures. This improvement enables WideResNet to capture complex patterns in image data more effectively. Leveraging the teacher model’s strong representation ability and the student model’s limitations in unseen regions further boosts the robustness and accuracy of anomaly detection.

## 5. Conclusions

This paper proposes a Synthetic-Anomaly Contrastive Distillation (*SACD*) framework for industrial image anomaly detection. *SACD* is built on two key components: (1) a reverse distillation framework where a pre-trained teacher network extracts hierarchical representations, guiding the student network with an inverse architecture to achieve feature alignment across multiple scales; and (2) *FeaCali* modules that refine the student’s outputs by filtering out anomalous feature responses. During training, *SACD* employs a dual-branch strategy, with one branch encoding multi-scale features from defect-free images and a Siamese anomaly branch processing synthetically corrupted samples. The *FeaCali* modules are trained to eliminate anomalous patterns in the anomaly branch, enabling the student network to focus exclusively on modeling normal patterns. A dual-objective optimization framework, combining cross-model distillation loss and intra-model contrastive loss, is used to train *SACD*, ensuring effective feature alignment and enhanced discrepancy amplification. At the inference stage, pixel-wise anomaly scores are computed based on discrepancies between the teacher’s representations and the student’s refined outputs across multiple layers. Extensive evaluations on the MVTec AD benchmark confirm our approach is effective and achieve superiority to current KD-based approaches for anomaly detection.

In future work, we plan to explore cross-domain anomaly detection scenarios, where the model trained on one type of industrial product is required to generalize to unseen categories or domains with minimal adaptation. This is crucial for practical deployment, as collecting labeled anomaly data for every product line is often infeasible. In addition, we aim to investigate more natural and physically consistent anomaly synthesis methods, beyond procedural noise, to better mimic the texture, geometry, and defect formation process of real-world industrial anomalies. Such approaches could further enhance the realism and diversity of training data, thereby improving the robustness of detection models in complex environments.

## Figures and Tables

**Table 1:** Anomaly detection results on MVTec AD [17]. For each category, the methods that achieved the top AUROC (%) are highlighted in bold. Our method ranks first based on the average scores of textures, objects, and overall performance.

Category/Method		US	MKD	MaMiNet	Patch-SVDD	PaDiM	CutPaste	RD4AD	AEKD	MMR	MSFR	Ours
Textures	Carpet	69.5	79.3	91.6	92.9	99.8	93.9	99.0	99.0	99.6	99.8	**100 ± 0.00**
	Grid	81.9	78.0	81.0	94.6	96.7	**100**	**100**	**100**	**100**	**100**	**100 ± 0.00**
	Leather	81.9	95.1	88.2	90.9	**100**	**100**	**100**	**100**	**100**	**100**	**100 ± 0.00**
	Tile	91.2	91.6	99.1	97.8	98.1	94.6	99.4	99.6	98.7	99.2	**99.8 ± 0.02**
	Wood	72.5	94.3	97.7	96.5	99.2	99.1	99.3	**99.8**	99.1	99.3	99.7 ± 0.03
	*Average*	*79.4*	*87.7*	*91.5*	*94.5*	*98.8*	*97.5*	*99.5*	*99.7*	*99.5*	*99.7*	***99.9 ± 0.01***
Objects	Bottle	91.8	99.4	99.0	98.6	99.9	98.2	99.8	**100**	**100**	**100**	**100 ± 0.00**
	Cable	86.5	89.2	86.2	90.3	92.7	81.2	96.9	**98.5**	97.8	97.5	**98.5 ± 0.06**
	Capsule	91.6	80.5	86.1	76.7	91.3	**98.2**	96.9	95.8	96.9	96.9	**98.2 ± 0.05**
	Hazelnut	93.7	98.4	93.1	92.0	92.0	98.3	**100**	**100**	**100**	**100**	99.2 ± 0.04
	Metal Nut	89.5	73.6	82.0	94.0	98.7	99.9	**100**	99.4	99.9	99.5	99.5 ± 0.03
	Pill	93.5	82.7	87.9	86.1	93.3	94.9	96.8	97.2	98.2	97.8	**98.0 ± 0.07**
	Screw	92.8	83.3	54.9	81.3	85.8	88.7	**98.8**	95.7	92.5	94.8	98.6 ± 0.04
	Toothbrush	86.3	92.2	95.3	100	96.1	99.4	98.6	**100**	**100**	98.9	**100 ± 0.00**
	Transistor	70.1	85.6	81.8	91.5	97.4	96.1	96.0	99.4	95.1	95.0	**99.7 ± 0.02**
	Zipper	93.3	93.2	91.9	97.9	90.3	**99.9**	97.7	98.8	97.6	97.6	99.0 ± 0.03
	*Average*	*88.9*	*87.8*	*85.8*	*90.8*	*93.8*	*95.5*	*98.2*	*98.5*	*97.8*	*97.8*	***99.1 ± 0.03***
*Total Average*		*85.7*	*87.8*	*87.7*	*92.1*	*95.4*	*96.2*	*98.6*	*98.9*	*98.4*	98.4	***99.3 ± 0.02***

**Table 2:** Anomaly localization results on MVTec AD [17] include AUROC (%) and PRO (%). AUROC is a pixel-wise comparison, while PRO is region-based. The best results in each item in bold.

Category/Method		US	SPADE	PaDiM	RIAD	CutPaste	RD4AD	AEKD	MMR	MSFR	Ours
Textures	Carpet	-/87.9	97.5/94.7	**99.1**/96.2	96.3/-	98.3/-	99.0/96.9	99.0/97.3	98.8/96.6	98.6/96.7	**99.1 ± 0.04**/**97.4 ± 0.10**
	Grid	-/95.2	93.7/86.7	97.3/94.6	98.8/-	97.5/-	99.3/97.7	99.3/97.6	99.0/96.5	98.8/96.6	**99.4 ± 0.03**/**97.8 ± 0.08**
	Leather	-/94.5	97.6/97.2	99.2/97.8	99.4/-	**99.5**/-	99.4/99.1	**99.5**/**99.2**	99.2/98.6	99.2/98.8	**99.5 ± 0.02**/**99.2 ± 0.03**
	Tile	-/94.6	87.4/75.9	94.1/86.0	89.1/-	90.5/-	95.7/90.8	96.1/92.5	96.6/90.2	95.4/90.3	**96.3 ± 0.08**/**92.2 ± 0.15**
	Wood	-/91.1	88.5/87.4	94.9/91.1	85.8/-	95.5/-	95.5/90.9	96.2/92.6	94.8/88.9	94.6/89.5	**96.5 ± 0.07**/**91.9 ± 0.18**
	*Average*	*-/92.7*	*92.9/88.4*	*96.9/93.2*	*93.9/-*	*96.3/-*	*97.8/95.1*	*98.0/95.8*	*97.7/94.3*	*97.3/94.4*	***98.2 ± 0.05**/**95.7 ± 0.11***
Objects	Bottle	-/93.1	98.4/95.5	98.3/94.8	98.4/-	97.6/-	98.7/96.7	99.0/97.1	98.3/96.0	98.2/95.3	**99.1 ± 0.02**/**97.2 ± 0.04**
	Cable	-/81.8	97.2/90.9	96.7/88.8	84.2/-	**90.0**/-	97.4/91.1	97.8/92.6	95.4/87.2	96.0/87.9	98.0 ± 0.06/**93.1 ± 0.08**
	Capsule	-/96.8	**99.0**/93.7	98.5/93.5	92.8/-	97.4/-	98.7/96.0	98.6/94.5	98.0/94.5	98.3/95.6	98.8 ± 0.03/**96.6 ± 0.05**
	Hazelnut	-/96.5	**99.1**/95.4	98.2/92.6	96.1/-	97.3/-	99.0/95.5	98.4/95.5	98.5/91.2	98.4/91.2	98.5 ± 0.04/**95.6 ± 0.06**
	Metal Nut	-/94.2	98.1/**94.4**	97.2/85.6	92.5/-	93.1/-	97.3/92.4	98.1/93.3	95.9/88.6	96.5/89.2	**98.3 ± 0.05**/93.1 ± 0.04
	Pill	-/96.1	96.5/94.6	95.7/92.7	95.7/-	95.7/-	98.1/96.5	98.2/96.7	98.4/96.1	98.9/96.6	**98.3 ± 0.04**/**96.7 ± 0.06**
	Screw	-/94.2	98.9/96.0	98.5/94.4	98.8/-	96.7/-	99.6/98.2	99.2/96.9	99.5/97.6	99.5/98.5	**99.7 ± 0.02**/**98.0 ± 0.03**
	Toothbrush	-/93.3	97.9/93.5	98.8/93.1	98.9/-	98.1/-	99.1/94.5	99.0/93.5	98.4/93.0	98.2/93.1	**99.3 ± 0.03**/**93.8 ± 0.07**
	Transistor	-/66.6	94.1/87.4	**97.5**/84.5	87.7/-	93.0/-	91.9/77.3	94.8/85.6	90.2/79.1	90.8/79.6	95.4 ± 0.09/**83.9 ± 0.20**
	Zipper	-/95.1	96.5/92.6	98.5/95.9	97.8/-	**99.3**/-	98.3/**95.6**	97.8/94.2	98.0/95.0	98.8/96.9	98.5 ± 0.03/95.8 ± 0.05
	*Average*	*-/90.8*	*97.6/93.4*	*97.8/91.6*	*94.3/-*	*95.8/-*	*97.8/93.4*	*98.1/94.0*	*97.1/91.9*	*97.4/92.4*	***98.4 ± 0.04**/**94.4 ± 0.09***
*Total Average*		*-/91.4*	*96.5/91.7*	*97.5/92.1*	*94.2/-*	*96.0/-*	*97.8/94.0*	*98.1/94.0*	*97.2/92.6*	*97.3/93.1*	***98.3 ± 0.04**/**94.8 ± 0.08***

**Table 3:** Anomaly localization results in terms of pixel-level AUROC on BTAD [46].

Method	Class 01	Class 02	Class 03	Average
VT-ADL	76.3	88.9	80.3	81.8
FastFlow	95	96	99	97
Patch-SVDD	94.9	92.7	91.7	93.1
RD4AD	**96.6**	96.7	99.7	97.7
Ours	96.5 ± 0.10	**97.4 ± 0.06**	**99.8 ± 0.02**	**97.9 ± 0.04**

**Table 4:** Comparison of model complexity and performance between *SACD* (under different *FeaCali* depths) and AEKD. Metrics include parameter count, FLOPs, inference latency per 256 × 256 image, and image-level AUROC on MVTec AD.

Depth (L)	Params (M)	FLOPs (G)	Inference Latency (ms)	Image-Level AUROC (%)
1	165.08	43.30	3.96	99.2
2	158.90	39.67	3.85	99.3
3	159.66	40.13	3.92	99.3
4	159.27	39.90	3.92	99.0
AEKD	132.92	33.98	4.64	98.90

**Table 5:** Comparison of model performance under various anomaly synthesis modes.

Description	Image-Level AUROC	Pixel-Level AUROC	PRO
CutPaste	99.1	97.9	94.2
Gaussian noise	99.0	98.0	94.4
Simplex noise	99.3	98.3	94.8

**Table 6:** Effect of hyperparameter α in the loss function.

α	Image-Level AUROC	Pixel-Level AUROC	PRO
0.01	99.0	98.3	93.9
0.1	99.2	98.2	94.2
0.2	99.3	98.3	94.8
0.3	98.9	97.9	93.6
0.5	98.5	97.4	93.5

**Table 7:** Comparison of model performance on different pretrained model.

Pretrained Model	Image-Level AUROC	Pixel-Level AUROC	PRO
ResNet18	98.4	97.6	94.0
ResNet50	99.0	98.0	94.4
WideResNet50	99.3	98.3	94.8

## Data Availability

Code will be released upon publication.
